# The impact of psychosocial adversity on brain and behaviour: an overview of existing knowledge and directions for future research

**DOI:** 10.1038/s41380-024-02556-y

**Published:** 2024-04-24

**Authors:** Nilakshi Vaidya, Andre F. Marquand, Frauke Nees, Sebastian Siehl, Gunter Schumann

**Affiliations:** 1 Centre for Population Neuroscience and Stratified Medicine (PONS), Department of Psychiatry and Clinical Neuroscience, Charité Universitätsmedizin Berlin, Berlin, Germany; 2 Donders Institute for Brain, Cognition and Behavior, Radboud University Nijmegen, Nijmegen, The Netherlands; 3 Institute of Medical Psychology and Medical Sociology, University Medical Center Schleswig Holstein, Kiel University, Kiel, Germany; 4 Centre for Population Neuroscience and Stratified Medicine (PONS), Institute for Science and Technology of Brain-Inspired Intelligence (ISTBI), Fudan University, Shanghai, China

**Keywords:** Neuroscience, Psychology

## Abstract

Environmental experiences play a critical role in shaping the structure and function of the brain. Its plasticity in response to different external stimuli has been the focus of research efforts for decades. In this review, we explore the effects of adversity on brain’s structure and function and its implications for brain development, adaptation, and the emergence of mental health disorders. We are focusing on adverse events that emerge from the immediate surroundings of an individual, i.e., microenvironment. They include childhood maltreatment, peer victimisation, social isolation, affective loss, domestic conflict, and poverty. We also take into consideration exposure to environmental toxins. Converging evidence suggests that different types of adversity may share common underlying mechanisms while also exhibiting unique pathways. However, they are often studied in isolation, limiting our understanding of their combined effects and the interconnected nature of their impact. The integration of large, deep-phenotyping datasets and collaborative efforts can provide sufficient power to analyse high dimensional environmental profiles and advance the systematic mapping of neuronal mechanisms. This review provides a background for future research, highlighting the importance of understanding the cumulative impact of various adversities, through data-driven approaches and integrative multimodal analysis techniques.

## Introduction

The interaction between individuals and their environment is a dynamic process, that occurs at multiple levels, including macro and micro-environment. While macroenvironment encompasses broad factors at neighbourhood level, the microenvironment refers to immediate surroundings and contexts in which individuals live their lives. Throughout life, individuals are exposed to multiple adverse events, within their microenvironment, that may create a cumulative burden of adversity, known as allostatic load [1, 2]. When faced with adversity physiological regulatory systems, such as the hypothalamic-pituitary-adrenal (HPA) axis, the autonomic nervous system (ANS), the metabolic system as well as the immune system, produce physiological response. While these responses are adaptive in the short term, repeated exposure to adverse events or chronic stress can lead to long-lasting alterations in these systems. This results in a so-called wear-and-tear or allostatic load which is known to significantly contribute to the emergence and maintenance of mental and physical illnesses [3]. According to its systemic cascades, the brain represents one of the critical targets for allostatic load. For example: chronic release of glucocorticoids or inflammatory cytokines can lead to changes in the brain [1, 2].

The human brain is well known to exhibit plasticity, an intrinsic ability to reorganise its structure and function throughout the lifespan [4]. This allows the brain to adapt to changes in the external environment or internal milieu. With respect to external environmental influences, like the experience of adverse events, the brain plays a central role in the processes of allostasis. Allostatic changes can lead to both successful adaptation and the development of resilience as well as dysfunctional behaviours and the emergence or maintenance of disorders [1–3]. In the latter case, the brain becomes vulnerable to dysregulation, leading to alterations in response to prolonged or severe adverse events.

Adversity can manifest in various forms including psychosocial factors such as childhood maltreatment, peer victimisation, social isolation, affective loss, domestic conflict, or poverty, as well as exposure to environmental toxins. Despite the shared aspect of stress biology underlying these adversities, they may still engage both common and distinct mechanistic pathways. The current understanding of these adverse events, however, is limited to a largely unidimensional perspective, with individual studies often focusing on isolated events [5–9]. While the nature of the impact is interconnected, the empirical evidence regarding the neurobehavioral effects from combination of different adversity types remains limited. It is crucial to move beyond the study of isolated events and investigate the cumulative/interactive effect or allostatic load, resulting from exposure to multiple adverse events, both simultaneously and successively, i.e., mirroring the complex and interconnected nature of real-life situations.

To gain insights into the effects of multiple adverse events, we can leverage data from existing large cohorts. In recent years, several large, well-phenotyped cohorts have emerged like IMAGEN [10], ABCD [11], cVEDA [12, 13], CHIMGEN [14], Generation R [15], ALSPAC [16, 17], UK Biobank [18], etc. Although they have been initiated under partly different research foci, they all encompass comprehensive information on the experiences of various adverse life events. These cohorts offer an opportunity to address the power limitations often encountered in smaller-scale studies and together with their multi-modal assessment batteries facilitate the examination of the combined effects of multiple exposures. By employing data-driven analyses in these large datasets, we can gain a deeper understanding of the intricate neurobiological effects of allostatic load. Finally, by encompassing diverse populations across cohorts, population neuroscience research can inform global precision psychiatry.

The objective of this review is to provide a comprehensive understanding of changes in brain structure and function following experiences of adverse life events. We have included those adversities that have been extensively investigated in the field of neuroscience. The choice was driven by both the prevalence of these experiences and their documented impact on brain across diverse populations. In the future perspective, we advocate for data-driven approaches to understand the cumulative impact of adversities, that can extend beyond the confines of selected categories. The exposures we targeted included childhood maltreatment, peer victimisation, loneliness, affective loss, domestic conflict, poverty, and toxins. Using title/abstract text words we combined these exposures with neuroimaging (MeSH) and human filter. The search was carried out in PubMed, covering the period from January 1, 2010, to April 8, 2023. The reference list of relevant systematic reviews identified in our structured search were hand-searched for relevant literature. In case of recent reviews/meta-analysis, direct citations were included in the manuscript. We exclusively reviewed cohort studies with participants recruited from the general population, deliberately excluding clinical samples, giving examples from cohorts with larger sample sizes (*n* > 300) and/or longitudinal designs, where possible. We highlight the extensive research conducted on various stressors at the individual level, summarising all identified papers in tables and representative examples in text; and emphasise the necessity of data-driven, multimodal approaches to better understand the complex relationships between multiple life events and brain. Lastly, we provide an outlook presenting an overview of various statistical approaches that can be employed in future studies.

### Childhood maltreatment

Current evidence suggests that negative experiences in the form of abuse and neglect during sensitive developmental periods can result in neuroplastic processes, disrupting normal brain functioning [5]. Studies among children, adolescents and adults with adverse childhood experiences all have reported detrimental effect of such experiences in multiple brain regions (see Table 1 for details). Specifically, the amygdala, the hippocampus, and the medial pre-frontal cortex (mPFC) are implicated, possibly due to the presence of dense glucocorticoid receptors and the timing for pathway development during childhood [1, 19, 20]. Decreased amygdala and mPFC volumes were linked to childhood abuse and decreased dorsolateral prefrontal (dlPFC) volume to childhood neglect [21–24]. Decreased hippocampal volume was linked to both childhood abuse as well as neglect [21, 25, 26]. In a study investigating the impact of childhood sexual abuse on brain development [27], sexual abuse was associated with lower hippocampal volume during childhood (3–5 and 11–13 years), but with lower frontal cortex volume during adolescence (14–16 years). The impact of childhood maltreatment may therefore depend on the type of adversity, in combination with a variation across different stages of development, potentially influencing different cognitive and emotional processes.

**Table 1:** Neuroimaging studies of childhood maltreatment.

Study, location	Participants	Mean age (SD; range) % Female	Exposure	Study design; covariates	Primary findings
**Childhood maltreatment**					
Purcell et al. [177] United Kingdom	300 participants from Birmingham Metropolitan area cohort	20 (1.5; 17–23) 50.3%	Childhood Trauma Questionnaire	Cross sectional Covariates: violence exposure, race, sex, and scanner type.	Stress-elicited ventromedial PFC, dorsolateral PFC, and hippocampal activity was lower in individuals who reported sexual abuse.
Silveira et al. [178] Canada	392 participants from National Consortium on Alcohol and Neurodevelopment in Adolescence (NCANDA)	17.36 (2.53; 12–22) 55%	Childhood Trauma Questionnaire	Cross sectional Covariates: age, sex, ethnicity, years of parental education, familial history of AUD, high-risk drinking, or frequent use of tobacco, marijuana, or other drugs	At baseline, distributed functional connectivity from hub regions in the bilateral dorsal anterior cingulate cortex, right anterior insula, right intraparietal sulcus, and bilateral pre- and postcentral gyri mediated the relationship between childhood trauma and executive dysfunction.
Puetz et al. [22] USA	414 participants from Duke Neurogenetics study (DNS)	19 (1; 18–22) 60%	Childhood Trauma Questionnaire	Cross sectional Covariates: age, sex, handedness, SES, psychopathology	Childhood abuse was linked to increased activity in the ventral amygdala, while neglect was linked to increased reactivity fronto-parietal network and dorsal amygdala.
Clausen et al. [132] USA	577 participants	32.25 (10.58; 18–59) 64%	Childhood Trauma Questionnaire	Cross sectional Covariates: age, sex, education, self-reported medical comorbidities, number of medications	Childhood trauma is associated with smaller regional GM volume within left superior frontal cortex and right medial cingulate cortex and higher regional GM volume within left medial cingulate cortex, right inferior insular cortex and left anterior insula.
Luo et al. [23] Europe	639 participants from IMAGEN 4121 participants from UK Biobank	IMAGEN: 19.06 (0.70) 50.8% UKB: 56.89 (5.02) 58.1%	Childhood Trauma Questionnaire	Cross sectional Covariates: sex, site, BMI PRS, family SES, stressful life events in the past year, birth weight, depressive symptoms, and illegal drug use	Childhood abuse was linked to obesity via prefrontal cortex.
Gheorghe et al. [40] United Kingdom	6751 participants from UK Biobank	62.1 (7.2; 45–80) 58.6%	Childhood Trauma Questionnaire	Cross sectional Covariates: age, sex, handedness, ethnicity, education, depression and anxiety and head size scaling	Childhood emotional abuse was associated with smaller cerebellar and ventral striatum volumes.
Ancelin et al. [179] France	398 participants from ESPRIT study	65–80 52%	Categorical presence of childhood abuse	Cross sectional Covariates: age, sex, brain volume, head injury, lifetime depression and anxiety disorder, psychiatric medication, and cardiovascular ischaemic pathologies.	Childhood adversity was associated with rostral middle frontal, lateral orbitofrontal, superior parietal, precuneus, and thalamus.
Koyama et al. [25] Japan	491 participants from Neuron to Environmental Impact across Generations (NEIGE) Study	65–84 52.7%	Categorical presence of childhood abuse	Cross sectional Covariates: age, sex, ICV, prescribed medication, smoking or drinking history, BMI, depressive and cognitive score, childhood and current SES, marital status.	Individuals with 2 or more adversities had larger anterior cingulate cortex and smaller amygdala and hippocampal volumes.
Korgaonkar et al. [180] Australia	647 participants	33.3 (12; 18.2–69.2) 51%	Categorical presence of childhood abuse	Cross sectional Covariates: age, sex, education, diagnosis, scan motion	Individuals who experienced abuse during childhood (but not during adolescence) had increased functional connectivity between brain networks involved in somatomotor processing and dorsal-ventral attention.
Cohen et al. [181] Australia	250 participants from Brain Research International Database (BRID)	39.9 (17.2; 18–70) NR	Early Life Stress Questionnaire	Cross sectional	Adverse childhood experiences were associated with anterior cingulate cortex and caudate nucleus.
Busso et al. [24] USA	51 participants from a longitudinal cohort	Baseline: 15.14 (1.46) measured adversity MRI at FU1: 16.96 (1.51) Clinical assessment at FU2: 18.92 (1.50)	Childhood Trauma Questionnaire	Longitudinal Covariates: age, sex, parental education	Childhood abuse was associated with reduced cortical thickness in vmPFC, right inferior frontal gyrus, left and right parahippocampal gyri, right inferior temporal gyrus, and right middle temporal gyrus.
Hanson et al. [39] USA	106 participants from a longitudinal cohort	Baseline: 13.67 (11.88–15.45) Follow up: 13.77–18.25 48.1%	Childhood Trauma Questionnaire	Longitudinal Covariates: age, time between scans, sex, depressive and anxiety symptoms.	Emotional neglect was associated with blunted development of reward-related ventral stratum activity.
Hein et al. [35] USA	167 participants from Fragile Families and Child Wellbeing Study (FFCWS)	Adversity at ages 3, 5, 9 MRI at 15 53.9%	Parent-Child Conflict Tactics Scale Mother’s report on partner/community violence and support	Longitudinal Covariates: sex, internalising psychopathology, and current life stress	Childhood violence exposure was associated with increased amygdala activation to angry faces in adolescence, whereas childhood deprivation was associated with decreased ventral striatum activation to happy faces in adolescence.
Goetschius et al. [182] USA	178 participants from Fragile Families and Child Wellbeing Study (FFCWS)	Adversity at ages 3, 5, 9 MRI at 15 56%	Parent-Child Conflict Tactics Scale Mother’s report on partner/community violence and support	Longitudinal Covariates: sex, race, pubertal development, adolescent life stress, maternal educational level and marital status at the child’s birth	Childhood violence exposure was associated with reduced rsFC density, with fewer salience network connections and salience network-default mode connections.
Ganella et al. [42] Australia	91 participants from a longitudinal cohort	Adversity measures at 15.02 (0.43; 13–15) MRI at 16.45 (0.51; 13–15) and 18.80 (0.44; 17–20) years 46%	Childhood Trauma Questionnaire	Longitudinal Covariates: age, sex, SES, diagnosis	Childhood maltreatment was associated with accelerated pituitary gland development in females.
Paquola et al. [26] Australia	123 participants Follow Up: 52 participants	19 (3; 14–28) 64.2%	Childhood Trauma Questionnaire	Longitudinal	Childhood maltreatment was associated with significantly stunted right hippocampal growth.
Farrow et al. [43] Australia	129 participants from Families and Childhood Transitions Study (FACTS)	Baseline: 8.4 (8–9.09) Follow Up: 9.9 (9.4–11.1) 52.7%	Lifetime Incidence of Traumatic Events Multidimensional Neglectful Behaviour Scale	Longitudinal Covariates: age, sex, SES, ICV	Childhood neglect was associated with greater baseline anterior pituitary volume, that was stable over the follow-up period.
Gehred et al. [183] New Zealand	861 participants of the Dunedin Study	Adversity accessed 7 times between 3 to 15 years. At 38 years retrospective account of adversity. MRI at 45 years. 49.3%	CDC–Kaiser Permanente Adverse Childhood Experiences Questionnaire	Longitudinal Covariates: Prenatal complications, Neurodevelopmental differences, perceived adult stress.	Childhood adversity, both prospectively and retrospectively accessed, was linked to smaller total surface area, thinner average cortex and smaller subcortical GMV. Stronger and more widespread association was observed for prospectively ascertained childhood adversity.
Hidalgo et al. [184] Netherlands	2993 participants from Generation R cohort	10.1 (0.6; 8.72–11.9) 50.8%	Life events and difficulty schedule.	Longitudinal Covariates: age at MRI, sex, total intracranial volume, maternal national origin, highest household education, and maternal prenatal alcohol use and smoking.	Childhood adversities (but not prenatal adverse events experienced by the mother) were related to global brain volume differences at age 10 years
**Institutionalised care**					
Tottenham et al. [48] USA	38 PI, 40 control	8.91 (2; 4.9–15.7) 25%	Institutionalised children	Covariates: age, cortical size	Late adoption was associated with larger corrected amygdala volumes, poorer emotion regulation.
Olsavsky et al. [49] USA	33 PI, 34 control	10.5 (3.5; 4–17) 46.5%	Institutionalised children	Covariates: age at adoption, age at scan, IQ	Previously institutionalised exhibited reduced amygdala discrimination between mothers and strangers. These effects correlated with age-at-adoption.
Herzberg et al. [50] USA	44 PI, 30 control	12.93 (0.58; 11.75–14.09) 67.5%	Institutionalised children	Covariates: age, sex, IQ, ICV	Later-adopted participants had decreased prefrontal volume and made fewer risky decisions.
Hodel et al. [47] USA	110 PI, 62 control	13.01 (0.55; 12.04–14.15) 66.3%	Institutionalised children	Covariates: age, sex, ICV	Hippocampal volumes showed an association with duration of institutional care, with later-adopted children showing the smallest volumes.
Sheridan et al. [51] Romania	136 PI, 72 control from Bucharest Early Intervention Project	Institutionalised: 0.5–2.75 Assessment: 11.14–14.68 49.6%	Institutionalised children	Covariates: age, sex	Prolonged institutional rearing leads to deficits in reward responsivity and implicit learning.

Childhood maltreatment including institutional rearing is known to have detrimental effects on brain development and functioning.

Neuroimaging studies at the functional level (fMRI) have mainly reported alterations in the amygdala, but findings are inconsistent [21]. One study on self-reported exposure to adversity suggests that the distinct pattern of amygdala activation depends on the timing of exposure during sensitive periods of development [28]. In this study, adversity reported during early childhood (3–6 years) was associated with blunted amygdala response, in contrast, exposure to adversity during early adolescence (13–15 years) was associated with an augmented amygdala response. In another study, differences within specific areas of amygdala, were attributed to the type of adversity [22]. Childhood abuse was linked with heightened reactivity in the ventral region of the amygdala, whereas experiences of neglect to heightened reactivity in the dorsal region of the amygdala.

Further, studies focussing on the frontal-limbic pathways also show converging effects on the amygdala [19, 29], reporting accelerated amygdala and mPFC connectivity in response to early adversity [30, 31]. These changes support behavioural research for increased threat processing i.e., inability to differentiate between safe and threatful stimuli, as form of adaptation [32] and provide backing to the stress acceleration hypothesis [33], i.e., early adversity expediting the development of the emotional regulation neural pathways. fMRI studies on emotional regulation following childhood maltreatment, also report alterations in the connectivity and activity of neural circuits in the frontal-limbic regions, more specifically in the amygdala and ventral anterior cingulate cortex (ACC) [34]. Differences in impact due to adversity type was again reported, in a longitudinal study, where childhood abuse was associated with increased amygdala activity while childhood neglect with decreased ventral striatum response to happy faces, in adolescents [35]. Other frontal-limbic regions beyond the amygdala that play a crucial role in the cognitive modulation of emotions like the dlPFC [36], and, in the automatic regulation of stress hormones like the hippocampus [37], are also implicated. However, the exact nature and direction of these functional changes have varied across studies [38].

In fMRI studies on reward processing, higher activation in the striatum among adolescents who have experienced childhood adversity is consistently reported [38, 39]. Additionally, adults who have experienced childhood emotional abuse exhibit reduced grey matter volume (GMV) in the ventral striatum [40]. Overall, a reduced anticipatory response to rewards is observed [29], which may represent an adaptive regulation towards avoidant responses during approach-avoidance conflict situations. This adaptive regulation is believed to increase the likelihood of survival in adverse environments. However, it can also hinder exploratory behaviour making it difficult to identify sources of reward in new environments [38, 41].

Other consistent findings include the effects on the pituitary gland, with longitudinal studies reporting accelerated development in response to childhood neglect [42, 43]. Lower superior parietal volume [21] and hyperactive superior temporal gyrus [44], linked to social perception and social cognition respectively, were also consistently reported. According to Nelson et al. [45], social information travels from the detection node to the affective node and then to the cognitive-regulation node. The impairments observed in all these networks, together reflect the complex interplay between early adversity, brain development, and resulting neurobiological changes.

### Institutionalised care

Children fostered from institutions experience lack of individual attention and have minimal opportunities for cognitive and social stimulation during crucial early years of development, when neural systems are highly plastic. Studies examining the effects of institutionalised care have found that these children exhibit smaller total GMV, reduced cortical thickness, impaired executive function skills, and atypical reward processing [46]. The availability of information on the known duration of institutionalised care provides a unique opportunity for investigating the precise relationship between the duration of adversity and its impact on neural outcomes. Here, we specifically review studies investigating the duration of institutionalised care (details in Table 1) to gain insights into the effects of temporally discrete adversity.

The duration of institutionalised care, as measured by the age at adoption, has been linked to both structural and functional brain alterations. Structural analyses have exhibited a dose-related relationship, where longer periods of time spent in foster care was associated with reduced hippocampus [47] and larger amygdala volumes [48]. Functional amygdala findings also showed an age-at-adoption relationship. Specifically, younger age-at-adoption was linked to more typical differentiation between mother and stranger stimuli, while older age-at-adoption was associated with reduced discrimination [49]. This struggle to differentiate between safe and dangerous stimuli, is reflective of an increased threat processing or fear generalisation. Further, institutionalised children exhibit altered risk-taking behaviour, with later adoptees making fewer risky decisions [50]. Prolonged institutionalisation is also associated with the potential for learning and adaptation in enriched environments post adaptation. In a notable study, children adopted after 5 years of institutionalisation failed to improve their performance in response to reward in a modified monetary incentive delay task at age 12, while those adapted by 2 years on average did improve [51].

These findings underscore the importance of capturing the duration of adversity. Researchers studying the impact of childhood adversity have predominantly relied on questionnaires [such as the Childhood Trauma Questionnaire [52]] to assess various forms of adversity experienced during the first 18 years of life. While these questionnaires provide valuable information, there is a growing recognition of the need to incorporate more specific details regarding developmental timing. By understanding the timing of exposure, researchers can explore how specific developmental stages may shape the impact of adversities on various outcomes, such as cognitive, emotional, and social functioning. Additionally, it is also important to consider the duration of exposure. Adversities that persist over a prolonged period can have distinct effects on individuals' development and well-being compared to isolated experiences. Including measures of duration in data collection will allow for a more nuanced understanding of allostatic load.

### Peer victimisation

Peer victimisation associated with bullying refers to persistent and repeated instances of aggression or intimidation, which can include verbal or physical assaults, social exclusion or peer rejection, name-calling, and threats [53] and is linked with alterations in several brain regions (see Table 2 for details). Structural findings in large cohort of children who were frequently bullied, calculated based on separate reports by parents and teachers, exhibit greater cortical thickness of the fusiform gyrus [54], a brain region known for its involvement in facial processing. Structural difference in the striatum has also been consistently reported [7]. In a longitudinal study, adolescents who experienced chronic victimisation and had larger putamen (dorsal striatum) volume at age 14 and showed a more rapid decrease in putamen volume compared to their counterparts who experienced less victimisation [55]. Putamen is associated with regulation of risky behaviour and processing of rewards.

**Table 2:** Neuroimaging studies of social pain.

Study, location	Participants	Mean age (SD; range) % Female	Exposure	Study design; covariates	Primary findings
**Peer victimisation**					
Eckstrand et al. [185] USA	81 participants	17.42 (2.16; 15–22) 59%	Victimisation questionnaire	Cross sectional Covariates: age, head movement	Sexual orientation victimisation was related to higher medial prefrontal cortex activation.
Corr et al. [186] USA	73 participants	12.8 (2.2; 9–16) 42%	Juvenile Victimisation Questionnaire	Cross sectional Covariates: age, sex, medication, psychiatry diagnosis	Greater polyvictimization was associated with reduced functional connectivity between the default mode and salience network.
Telzer et al. [57] USA	38 females measured across 7 years	15.43 (0.33; 14.9–16.3)	Social Experiences Questionnaire-Revised	Longitudinal	Greater severity of peer victimisation was linked to heightened activation in the amygdala, ventral striatum, fusiform gyrus, and temporoparietal junction in response to in-group compared to out-group peers.
Telzer et al. [187] USA	46 females measured across 7 years	15.3 (0.34; 14.8–16.1)	Social Experiences Questionnaire-Revised	Longitudinal	Victimised girls showed greater reactivity in affective sensitivity, including the bilateral amygdala, ventral striatum, and orbitofrontal cortex as well as regions involved in social cognition, including the medial prefrontal, temporal parietal junction, and medical posterior parietal cortex.
Quinlan et al. [55] Europe	682 participants from IMAGEN cohort	Baseline: 14.4 (0.4) Follow-up 1: 16.5 (0.6) Follow-up 2: 19.0 (0.7) 54%	Revised Olweus Bully/Victim Questionnaire	Longitudinal Covariates: Sex, study site, SES, pubertal status, and change in intracranial volume	Chronic peer victimisation was associated with steeper decreases in left putamen volume.
Muetzel et al. [54] Netherlands	2602 participants from Generation R	Teacher report: 6.6 (4.6–9.6) Parent report: 8.1 (7.5–9.9) MRI: 10.09 (0.57; 8.5–11.9) 51%	Bullying Involvement Assessment	Longitudinal Covariates: Age, Sex, Ethnicity, maternal education, Child’s non-verbal IQ and psychiatric illness.	Children classified as frequent targets of bullying showed thicker cortex in the fusiform gyrus.
**Loneliness**					
Kong et al. [60] China	308 participants	19.94 (1.27; 18–27) 54.2%	UCLA loneliness scale	Cross sectional Covariates: age, sex, total GMV	Lonely individuals had greater regional grey matter volume in the left dorsolateral prefrontal cortex, which might reflect immature functioning in terms of emotional regulation.
Düzel et al. [59] Germany	319 participants from Berlin Aging Study	70.1 (3.7; 61–88) 50.9%	UCLA loneliness scale	Cross sectional Covariates: age, sex, education, social network size, depressive affect, openness, morbidity, total intracranial volume, time interval between MRI and assessment	Loneliness was associated with smaller GMV in the left amygdala/anterior hippocampus, left posterior parahippocampus, and left cerebellum.
Liégeois et al. [188] USA	419 participants from Human Connectome Project Replication in 328 adults	NR (22–35)	Loneliness survey from the NIH Toolbox on Emotion	Cross sectional Covariates: age, sex, race, motion, motion.	Static and dynamic FC explain loneliness equally well, while specifically dynamic FC encodes cognitive tasks like working memory.
Mwilambwe-Tshilobo et al. [64] USA	942 participants from Human Connectome Project	28.0 (3.5; 22–37) 53.7%	Loneliness survey from the NIH Toolbox on Emotion	Cross sectional Covariates: age, gender, MMSE, positive affect, and personality measures	Loneliness associated with dense, lower modularity (increased integration) between default, frontoparietal, attention and perceptual networks.
Tao et al. [61] USA	1829 participants from Framingham Heart Study	46.3 (8.6) 54%	One-item loneliness measure	Cross sectional Covariates: age, sex, education, and the time between assessments	Persistent loneliness was associated with smaller temporal lobe volume. Cumulative loneliness score was associated with smaller brain volumes in the hippocampus and with enlarged lateral ventricles.
Brilliant et al. [70] Japan	1336 participants	20.8 (1.7; 18–27) 43.48%	Revised UCLA loneliness scale	Cross sectional Covariates: age, sex	Loneliness was associated with higher functional connectivity between the inferior frontal gyrus and supplementary motor area, precentral gyrus, and superior parietal lobule.
Kiesow et al. [189] United Kingdom	10,129 participants from UK Biobank	55 (7.5; 40–69) 52.4%	One-item loneliness measure	Cross sectional	Greater volumetric deviations of the amygdala between lonely and non-lonely males compared to females and more volumetric deviations in ventromedial prefrontal cortex and visual sensory network in between lonely and non-lonely females compared to males.
Spreng et al. [63] United Kingdom	38,701 participants from UK Biobank	54.9 (7.5; 40–69) 56.38%	One-item loneliness measure	Cross sectional	Increased functional connectivity of the default mode network was observed in lonely individuals.
Velpen et al. [62] Netherlands	3,737 (cross-sectional), 3,720 (longitudinal) participants from Rotterdam Study	59.6 (8; 45.5–92.7) 54.7%	One-item loneliness measure	Longitudinal: Median follow up time 4.1 years. Covariates: age, sex, and total intracranial volume	Participants with better perceived social support had larger total brain volumes. They also had a less steep decline in total brain volume over time than those with suboptimal social support.
**Affective loss**					
Chen et al. [77]	56 participants	69.5 (8.61) 26%	Inventory of Complicated Grief	Longitudinal Covariates: age, sex, education and voxelwise grey matter concentration	Increased amygdala functional connectivity.
Perez et al. [88] Netherlands	5501 participants from Rotterdam cohort	61.55 (8.95) 53.8%	Inventory of Complicated Grief	Cross sectional Covariates: age, sex, education, systolic blood pressure, diabetes mellitus, history of stroke, depression and anxiety, current depressive symptoms and alcohol consumption.	Smaller brain volume.
Blair et al. [76] USA	66 participants	66.1 (8.9; 51–87) 69%	Inventory of Complicated Grief	Cross sectional Covariates: age, sex, time since loss, depressive score and TGMV.	Higher functional connectivity between ventral caudate and the medial prefrontal, orbitofrontal, and subgenual cingulate cortices.
Arizmendi et al. [78] USA	28 participants	71.9 (62–82) 81%	Inventory of Complicated Grief	Cross sectional	Complicated Grief showed an absence of increased rostral ACC (rACC) and fronto-cortical recruitment in emotional task.
Freed et al. [82] USA	20 participants	37.8 (13.1; 22–62)	Emotional Stroop task	Cross sectional	High DLPFC-amygdala connectivity correlated with reduced attentional bias, while low rACC-amygdala connectivity predicted sadness intensity.
Najib et al. [81] USA	11 females	25.9 (5.8)	Grief rating scale	Cross sectional	Grieving breakup was associated with altered activity in the cerebellum, anterior temporal cortex, insula, anterior cingulate, and prefrontal cortex.
Luo et al. [80] China	107 participants	56.96 (6.06) 57.9%	Loss of only child	Cross sectional Covariates: age, sex, ICV	Left hippocampal volumes were significantly smaller in individuals who lost their only child.
Kersting et al. [79] Germany	24 females	30.2 (5.1)	Perinatal Grief Scale	Cross sectional	Loss of an unborn child was closely related to the activation of the physical pain network encompassing the cingulate gyrus, the inferior frontal gyrus, the thalamus, and the brainstem.
Acosta et al. [87] Germany	196 participants	24 (3.2; 19–38) 50%	List of Threatening Experiences Questionnaire	Cross Sectional Covariates: anxiety and depression scores, attachment security and parental divorce in childhood and childhood maltreatment	Experience of at least one AL is associated with larger bilateral amygdala volumes, smaller right hippocampal volume.
Acosta et al. [86] Germany	192 participants	24.1 (3.2; 18–40) 50%	List of threatening experience questionnaire	Cross sectional Covariates: age, TICV	No significant association between AL and brain grey matter volume in the cerebellum.
Benetti et al. [85] United Kingdom	32 participants	25.2 (4.3) 53.1%	List of threatening experience questionnaire	Cross sectional Covariates: age, sex	Greater number of affective losses was associated with increased cerebellum volume.
**Domestic conflict**					
Chester et al. [91] USA	100 participants	21.61 (3.73; 18–35) 51%	Abuse within intimate relationships scale	Cross sectional	Partner aggression linked to greater connectivity between ventral and dorsal medial frontal cortex. Men had more neural response to provocation while women showed more neural response during aggression itself.
Tomoda et al. [190] USA	52 participants	21.7 (2.25; 18–25) 73%	Verbal Aggression Scale	Cross sectional Covariates: age, sex, level of parental verbal aggression, parental education, financial stress, full scale IQ, and total GMV	Witnessing domestic violence subjects had a 6.1% GMV reduction in right lingual gyrus. And reduced thickness in visual cortex and occipital pole.
Graham et al. [89] USA	24 infants	0.69 (0.15; 0.5–1) 33.3%	Revised Conflicts Tactics Scale	Cross sectional Covariates: age	Higher levels of interparental conflict were associated with greater activation to very angry tone of voice in the rostral ACC and subcortical structures, including the hypothalamus.
Flanagan et al. [191] USA	20 participants	39.45 (10.27; 22–58) 55%	Revised Conflict Tactics Scale	Cross sectional	Greater activation during the relationship conflict cue compared to the neutral cue in amygdala and prefrontal cortex
Roos et al. [90] South Africa	36 females	25.36 (6.16; 16–38)	Abuse Assessment Scale	Cross sectional Covariates: age, alcohol use, TICV	Altered connectivity on a global and regional level in the IPV group of regions involved in cognitive-emotional control.
Daugherty et al. [192] Spain	55 females	40.78 (12.43)	Composite Abuse Scale—Short Form	Cross sectional Covariates: alcohol and substance use, age, education.	Volume alterations in precuneus, superior occipital, superior temporal, opercular, transverse temporal, frontomarginal, temporal occipital were observed.

Peer victimisation, social isolation, affective loss, and domestic abuse contribute to the experience of social pain.

fMRI studies on these behaviours (risk-taking and reward processing), have had mixed results, as reported in a recent systematic review [7]. However, an interesting pattern was observed in relation with rejection sensitivity or need for belonging. A higher likelihood of engaging in risky behaviour is commonly observed during adolescence, especially in social situations involving peers. For example: in a driving stimulation task, an increased activation in the ventral striatum and OFC associated with risk-taking was observed in the presence of peers [56]. Thus, when peer evaluation or the need for belonging is present, there is a heightened sensitivity to the potential reward value of risky decision. In another study, female adolescents with a history of higher exposure to peer victimisation showed greater activation in the amygdala, ventral striatum, fusiform gyrus, and temporoparietal junction in response to in-group rather than out-group peers, indicating greater anticipation of reward and outcome value towards in-group peers [57]. These findings suggest that higher risk-taking behaviour could be a response to avoid peer rejection and thus highlight the need to study overt (physical) and covert (relational) victimisation separately.

Covert victimisation is linked to social pain, i.e., emotional distress or discomfort experienced due to negative social experiences, including rejection, exclusion, or betrayal. Social pain reported during exclusion task paradigms, is consistently associated with increased activation in ACC and insula [58]. Further, studies investigating neural correlates of social exclusion have consistently observed increased neural activity in regions associated with the processing of emotions, such as the amygdala, dorsolateral ACC, and inferior fusiform gyrus, in individuals who have experienced peer victimisation compared to those who have not [7]. Thus, peer victimisation might exacerbate the emotional pain experienced during social exclusion.

### Social isolation/loneliness

Loneliness, a complex socio-emotional trait is a strong predictor of mental illness. Loneliness has been associated with larger GMV of the dlPFC and smaller volume of amygdala, anterior hippocampus, posterior para-hippocampus, and cerebellum [59–61], interestingly in the left hemisphere for all aforementioned regions (details in Table 2). Further, loneliness is strongly correlated with perceived social support. A longitudinal investigation revealed that individuals who reported higher levels of perceived social support experienced less decline in total brain volume as opposed to those with suboptimal perceived social support [62]. Of note, in a large cohort study of middle-aged adults, enlarged ventricles were also associated with loneliness [61]. Thus, loneliness could result in steeper cognitive decline.

In resting-state data-driven analysis, loneliness was associated with increased functional connectivity (FC) of the default mode network, frontoparietal network and attention and perceptual networks [63, 64]. Hypervigilance and stress reactivity, which are believed to be associated with loneliness, may be connected to these networks. According to the loneliness model, social isolation leads to unconscious surveillance for social threats, i.e., implicit hypervigilance [65, 66] and is paired with attentional bias and confirmatory behaviour. Attentional bias is characterised by a heightened focus on negative social cues, which can contribute to feelings of rejection [67]. Confirmatory behaviour involves engaging in inappropriate social and withdrawal behaviours, which can elicit negative reactions and reinforce the initial negative beliefs about interpersonal interactions [68]. Together, this causes lonely individuals to perceive the social world as threatening and display negative social behaviour and affect [69]. This is further supported by a large cohort study [70] which showed increased FC between inferior frontal gyrus (IFG) with superior parietal lobule, precentral gyrus and supplementary motor area. Heightened inferior frontal connectivity is associated with selective attention and social cognition [71, 72], superior parietal lobule is associated with working memory, attention and visuospatial perception [73, 74], and precentral gyrus is associated with pain appraisal [75]. Thus, lonely individuals actively observe their environment, are cautious about negative cues with heightened threat appraisal.

### Affective loss

Separation, ending a significant relationship or death of loved one is categorised as affective loss. While transient subclinical symptoms of anxiety and depression might be present initially, many studies have demonstrated long-term clinical effects as well, referred to as prolonged grief disorder (PGD) [6]. We did not find any large cohort studies investigating FC associated with affective loss and have reviewed smaller studies to gather information on this topic. fMRI studies on bereavement of a first-degree relative [76, 77] or spouse [78], the loss of an unborn child [79] or only child [80], the breakup of a relationship [81] and the loss of a pet [82] have reported altered neural activations in the networks of pain and emotions, including the cingulate, amygdala, hippocampus, and OFC (details in Table 2). Functional studies of amygdala have reported heightened activation with dlPFC linked to attentional bias and with rostral ACC linked to emotional dysregulation [82]. The posterior cingulate cortex involved in autobiographic memory also shows heightened activation during grief [6]. Finally, repetitive thinking involving OFC was also observed [76].

However, the statistical power of reported studies has been limited due to small sample sizes which could potentially cause problems with replicability [83] and type I errors, i.e., risk of obtaining false positive results [84]. For example: while a small study (*n* = 32) reported increased GMV in cerebellum [85], results were not replicated in a larger (*n* = 192) sample [86]. Studies on structural MRI though few, on the other hand have recruited larger sample (*n* ~ 190) [86, 87] or are nested in cohort: Rotterdam cohort (5501 participants) [88]. These studies associate affective loss with smaller GMV (246), specifically in the amygdala [87].

### Domestic conflicts

Exposure to domestic conflicts, whether as a witness or a victim of abuse, is highly distressing and potentially traumatic. Maternal reports of higher interparental conflict have been linked with increased neural responses in infants to highly angry speech compared to neutral speech. This hyperactivation was observed in several brain regions involved in emotional processing and stress reactivity, such as the rostral ACC, caudate, thalamus, and hypothalamus [89]. Interparental conflict may have an impact on early emotional development due to poor caregiving and via direct exposure to aggressive interactions between caregivers, leading to challenges in the emotional regulation within this dyadic relationship.

Victims of intimate partner violence (IPV) are known to adapt their emotional regulation strategies to manage recurring stressful events, thus affecting their underlying brain connectivity. To get an overview of the brain correlates of IPV, we again looked at smaller population-based studies (Table 2 for details). Structural network connectivity study shows altered connectivity in the victim group, in regions involved in cognitive-emotional control. Specifically, the caudal ACC, middle temporal gyrus, left amygdala, and ventral diencephalon (including the thalamus) were implicated in these alterations [90]. A study on laboratory-based and real-world intimate partner aggression (IPA) showed distinct patterns in males and females. Specifically, men’s IPA was associated with reduced reactivity in the posterior cingulate during provocation, while women’s IPA was associated with decreased activity in the ventromedial prefrontal cortex during aggressive event [91]. However, due to the cross-sectional nature of these studies, it is not possible to establish a causal pathway.

### Poverty

Socioeconomic status (SES) as a comprehensive measure includes various aspects of sustained experience, incorporating both objective factors such as education and income, as well as subjective factors such as social standing. SES influences an individual’s environment throughout their lifespan and is associated with experience-based neural plasticity [4]. The environmental stimulation, or lack thereof in impoverished conditions, has a potential effect on the brain. Brain structure and functional connectivity are associated with SES. However, these associations do not converge on specific regions or networks and are more widespread (which could also be a result of varied assessments). Whole brain structural studies reporting positive association between SES and global brain measures including cortical thickness, cortical surface area and GMV [92–95] reflect the global effects of SES. Lower SES is further associated with lower GMV in specific regions such as the amygdala, hippocampus, striatum, thalamus, cingulate cortex, occipital cortex, and frontal and temporal lobes [9, 96, 97]. These findings are replicated in large [92, 98], longitudinal [93, 95, 99] cohorts and are independent of genetic architecture [94] (details in Table 3). Taken together, these regions are responsible for language processing, reading skills, visuo-spatial abilities, decision-making and executive functioning.

**Table 3:** Neuroimaging studies of poverty.

Study, location	Participants	Mean age (SD; range) % Female	Exposure	Study design; covariates	Primary findings
Poverty					
Butterworth et al. [102] Australia	431 participants from PATH study	46.7 (0.07; 44–48) 65.8%	Financial hardship questions	Cross sectional Covariates: age, sex	Current financial hardship was associated with smaller left and right hippocampal and amygdala volumes
Chan et al. [103] USA	304 participants	20–59 60.7%	Family income	Cross sectional Covariates: age, sex, childhood SES, mental health and cognitive ability	Current SES was related to segregation of large-scale functional brain networks and thinner mean cortical grey matter.
Noble et al. [94] USA	1099 participants from PING study	11.9 (4.9; 3–20) 48.3%	Family income	Cross sectional Covariates: age, sex, scanner site, and genetic ancestry factor	In children from lower income families, even small differences in income were linked to significant differences in surface area, whereas in children from higher income families, similar income changes were associated with smaller differences in surface area.
Kim et al. [98] USA	7569 participants from the ABCD cohort	9.91 (0.52; 9–10) 47.5%	Income-to-needs ratio	Cross sectional Covariates: sex, race, parental educational level, study site, baseline psychiatric problems	Living in poverty was associated with less cortical surface area in the left superior temporal gyrus, left fusiform gyrus, right lateral occipital cortex, and right middle frontal gyrus. And smaller cortical volumes in the left superior temporal gyrus, postcentral gyrus, lateral occipital cortex, lateral orbitofrontal cortex, right lateral occipital cortex, transverse temporal gyrus, and rostral middle frontal gyrus.
White et al. [105] USA	172 participants	13.49 (0.52; 12–15) 65.7%	Income-to-needs ratio	Cross sectional Covariates: age, sex, race	Larger response in brain regions implicated in attention to reward and loss cues and to reward and loss feedback.
Javanbakth et al. [100] USA	52 participants from longitudinal cohort	23.6 (1.2; 22–25) 46.1%	Income-to-needs ratio	Longitudinal Covariates: age, sex	Childhood poverty, independent of concurrent adult income, was associated with higher amygdala and medial prefrontal cortical and with decreased left amygdala and medial prefrontal cortex functional connectivity.
Luby et al. [93] USA	145 participants from Preschool Depression cohort	9.78 (1.29; 6–12) 51%	Income-to-needs ratio	Longitudinal Covariates: age, sex, pubertal status, history of psychiatric disorders and psychotropic medication use	Poverty was associated with smaller cortical grey matter and hippocampal and amygdala volumes.
Hair et al. [99] USA	389 participants from Normal Brain Development cohort	12 (4–22) 52.5%	Income-to-needs ratio	Longitudinal Covariates: birth weight, race, family size, and maternal education.	Children from poor families had structural differences in the frontal lobe, temporal lobe, and hippocampus.
Kim et al. [101] USA	49 participants from longitudinal cohort	Poverty measured at 9 years. MRI at 24	Family income	Longitudinal Covariates: Current income	Adults with lower family income at age 9 exhibited reduced ventrolateral and dorsolateral prefrontal cortex activity and failure to suppress amygdala activation at age 24.
Hair et al. [99] USA	486 participants from Normal Brain Development cohort	10.1 (5.54; 0–20) 51.9%	Income	Longitudinal Covariates: birth weight, site	Structural differences in grey matter development for children living in or near poverty, first detected during childhood (age 2.5–6.5 years), evolve throughout adolescence.
McDermott et al. [95] USA	623 participants from longitudinal cohort	First scan: 12 (4; 5.2–25.4) 47.9%	Hollingshead SES scale	Longitudinal Covariates: age, sex	Higher SES is associated with areal expansion of lateral prefrontal, anterior cingulate, lateral temporal, and superior parietal cortices and ventrolateral thalamic, and medial amygdalo-hippocampal subregions.

Poverty, as a socioeconomic factor, influences various proximal factors and can have cascading effects on brain development and functioning.

From studies on children and adolescents, one can reliably state that socio-economic disadvantage is linked to changes in overall cognitive development. Children living in poverty have a higher likelihood of encountering developmental delays, lower performance on cognitive and academic assessments, and an increased occurrence of behavioural and emotional issues compared to their more privileged counterparts [96, 97]. In one study, children living 1.5 times below the federal poverty line in the US had regional GMV that were 3–4 points lower than the developmental norm, defined by index of structural brain development based on full longitudinal study sample. Further, these lower volumes mediated the association between low-income status and scoring 4–7 points lower on standardised tests - the Wechsler Abbreviated Scale of Intelligence and Woodcock-Johnson III Tests of Achievement [92]. Another study reported that brain variations can be detected as early as 2.5–6.5 years [99]. In the temporal, parietal, and occipital lobes grey matter differences between children from low SES and those from higher SES were observed as early as 2.5 years of age, and these disparities persisted throughout the studied age range up to 20 years. While differences in GMV in the frontal lobe emerged later in development, around 6.5 years of age, and continued to intensify until 20 years.

Among adults, associations have been reported with childhood SES [100, 101] as well as current SES [102, 103]. Lower family income at age 9 was associated with reduced activity in the vlPFC and dlPFC and failure to suppress amygdala activation while regulating negative emotions in 24-year-olds [101]. Both the dlPFC and vlPFC play crucial roles in cognitive control and executive functioning, supporting the regulation of goal-directed behaviours. Decreased activity in these regions along with amygdala has been linked to disruptions in down-regulation of negative emotions [101]. Current SES in middle aged adults was associated with reduced hippocampus and amygdala volumes [102] and thinner average cortical grey matter [103] after controlling for childhood SES. Further, current SES moderated the association between age and brain system segregation [103]. Middle aged adults with lower SES showed decreased system segregation compared to those with higher SES. Taken together, these findings underscore the importance of SES as an important factor that influences individuals across the entire lifespan.

In fMRI studies lower SES has been linked to reduced activation of hippocampus and amygdala in resting-state fMRI [9]. In task-based fMRI, with working memory paradigm, reduced activation of frontal and temporal regions linked with cognitive functioning are observed for lower SES [9, 104]. Further, lower SES is associated with higher threat reactivity and risk aversive decision making linked to increased amygdala and mPFC, respectively [105]. In a longitudinal study on response inhibition, lower SES was linked to higher activation of ACC [106]. Finally, lower SES is associated with hypoactivation of the executive network and hyperactivation of the reward network [104].

fMRI studies on reward processing have also associated low SES with widespread neural correlates. Alterations in reward processing is linked to caudate/striatum and OFC and parietal cortex in MID task; the dorsomedial frontal, subgenual ACC, dlPFC, and parietal cortices in gambling task and the dorsomedial frontal cortex in guessing task [104]. Individuals who experience material deprivation may face challenges in optimising their rewards, as the costs associated with delayed rewards and missed opportunities are disproportionately higher for those with limited available resources [107].

Overall, a multitude of neural associations are found across various brain regions. A recent meta-analysis of structural and functional studies also supported this widespread associations [104]. SES is a multi-dimensional construct which is nested in an ecological system. Therefore, its effects must be comprehensively understood within the psychosocial context of the population being studied. Individuals from low SES experience a range of unfavourable psychosocial and physical conditions that occur together and are interrelated [108]. These conditions are often suboptimal and therefore underscore the impact of poverty. For example: A person’s exposure to familial violence and crime incidence in their neighbourhood is negatively correlated with household income. Similarly, social class is correlated with contact with aggressive peers [108, 109]. Further, low-income families may live in communities that have higher physical adversities. In the second article of this series (Polemiti et al. [110]), the review focuses on the physical environment at the community level, while the impact of neurotoxins at the individual level is discussed below. Overall, exposure to violence, inadequate cognitive stimulation or social support, and a range of other hindrances and discomforts make it challenging to establish a single and straightforward explanation for the associations with poverty. Therefore, it is possible that the widespread effects observed across various brain regions in different studies may be capturing complex underlying interactions among these factors and could be disentangled in future studies.

### Toxins

While neurotoxins are not strictly part of the microenvironment, they are environmental factors that individuals may be exposed to within their immediate surroundings. Humans are continuously exposed to a wide range of neurotoxins [111], including heavy metals or metalloids (e.g., arsenic, lead) [112–114] as well as man-made chemicals (e.g., polychlorinated biphenyls (PCBs), phthalates) [111, 115]. The timing and dosage of such exposure plays a crucial role and is associated with more pronounced effects [111]. Among all studies (details in Table 4), changes in GMV have been reported. This included changes as a consequence of arsenic exposure mostly through food or contaminated water [116], which is high in particular regions of the world, including South America, the United States and central Asia, but also due to exposure to lead, which is often used in various products, including pipes or gasoline [117], and finally due to pesticides, such as organophosphates, which are used to protect harvest or preserve food throughout the world and are highly toxic, especially to infants and children [111].

**Table 4:** Neuroimaging studies of toxins.

Study, location	Participants	Mean age (SD; range) % Female	Exposure	Study design; covariates	Primary findings
Vaidya et al. [118] India	1014 participants of Consortium-on-Vulnerability-to-Externalising-Disorders-and-Addictions (cVEDA)	14.9 (4.8; 6–23) 44.5%	Arsenic	Cross-Sectional	Higher arsenic exposure was associated with increased volume in inferior frontal cortex, and decreased volume in right inferior temporal cortex, right rostral anterior cingulate cortex, and left Insula. And alterations in functional brain activity in inferior frontal gyrus, insula, inferior temporal gyrus and rostral anterior cingulate cortex.
Suchy-Dicey et al. [193] United States	687 healthy adults; Strong Heart Study & Cerebrovascular Disease and its Consequences in American Indians (CDCAI)	(65–80) 68%	Arsenic, Cadmium	Cross-Sectional	Higher arsenic burden was associated with higher white matter hyperintensity.
Acosta-Cabronero et al. [194] Germany	116 healthy adults	54 (19; 20–79) 48%	Iron	Cross-Sectional	Higher iron concentration was associated with age-dependent clusters in sensory-motor, dosal-frontal lobes, posterior incular, cerebellum, dentate nucleus, putamen, caudate nucleus, thalamus, mammillary bodies.
Zachariou et al. [195] United States	95 healthy adults	69.7 (5.7; 60–86) 63%	Iron	Cross-Sectional	Higher iron concentration was associated with low neurite density within task-relevant white matter networks (especially within frontal and parietal cortical regions).
Beckwith et al. [196] United States	155 participants of Cincinnati Lead Study (CLS)	27 (1.1; 25–31) 58%	Lead (lead levels measured in pregnant mothers)	Cross-Sectional	Higher lead exposure in childhood was associated with lower grey matter volume in several regions in females (frontal gyri, temporal lobe) and males (predominantly frontal and parietal lobe).
Beckwith et al. [197] United States	123 participants of Cincinnati Lead Study (CLS)	27 (1.1; 27–33) 58%	Lead (lead levels measured in pregnant mothers)	Cross-Sectional	Higher lead exposure in childhood (at age 78 months) was associated with three grey matter clusters including the Cingulate, Medial Frontal Gyrus, Superior Frontal Gyrus, Paracentral Lobule, Supplementary Motor Area.
Marshall et al. [119] United States	9712 participants of participants of Adolescent Brain Cognitive Development (ABCD)	(9–10) 47.4%	Lead	Longitudinal	Higher risk of lead exposure in childhood was associated with smaller cortical volume and cortical surface area, especially in low-income households.
Marshall et al. [120] United States	8524 Adolescent Brain Cognitive Development (ABCD)	(9–10) 47.6%	Lead	Longitudinal	Higher risk of lead exposure in childhood was associated with smaller volume of the corpus callosum (mid-anterior, central, mid-posterior)
Schwartz et al. [122] United States	532 male participants of Former organolead workers	56.1 (7.7; 45–75)	Lead	Longitudinal	Higher occupational lead exposure (18 years ago) was associated with smaller total brain volume, as well as volume in regions of cingulate gyrus, insula and corpus callosum.
Reuben et al. [121] New Zealand	564 participants of Dunedin Study	45 46.5%	Lead	Longitudinal	Higher lead exposure at age of 11 years was associated with smaller cortical surface area, smaller hippocampal volume at age 45 years.
Migneron-Foisy et al. [198] Canada	89 participants of Nunavik Child Development Study (NCDS) birth cohort	18.4 (1.2; 16–22) 57%	Mercury, Polychlorinated Biphenyls (PCBs), Lead	Longitudinal	Higher differences in pre-postnatal mercury and polychlorinated levels were associated with higher fractional anisotrophy in several regions of the corpus callosum (anterior and posterior midbody, isthmus, splenium).
Invernizzi et al. [199] Italy	193 participants of Public Health Impact of Metals Exposure (PHIME) study	19.2 (15–25) 53%	Manganese, lead, copper, chromium	Cross-Sectional	Higher dosage of metal mixture was associated with lower global and local efficiency in rsMRI network (111 brain regions).
Van den Dries et al. [200] Netherlands	518 participants of Generation R	9.9 (9–12) 50.6%	Organophosphates	Longitudinal	10-fold increase in averaged dimethyl metabolite concentrations across pregnancy was associated with lower fractional anisotrophy and higher mean diffusivity in most tracts EXCEPT uncinate fasciculus, (left), forcepts major (right), corticospinal tract (right).
Binter et al. [123] France	95 participants of Perturbatuers endocriniens: Etude Longitudinale sur les Anomalies de la Grossesse, l’Infertilité et l’Enfance (PELAGIE)	10.8 (10–12) 56.8%	Organophosphate pesticides—dialkylphosphate (dap)	Longitudinal	Higher dialkylphosphate levels were associated with lower brain activity in left inferior frontal gyrus, bilateral superior frontal gyrus (during successful inhibition).
Lamoureux-Tremblay et al. [201] Canada	71 participants of Nunavik Child Development Study (NCDS)	18.3 (0.1; 16–22) 56%	Polychlorinated Biphenyls (PCBs), Mercury, lead	Longitudinal	Moderate to high concentration of polychlorinated biphenyls were associated with greater differential activation (during conditioning) in the right orbitofrontal cortex. And lower differential activation (during extinction) in right anterior cingulate cortex, higher differential activation in right dorsolateral prefrontal cortex.
Sussman et al. [202] Canada	46 participants of GEStation and the Environment cohort (GESTE)	10.4 (0.6; 9–12) 37%	Polychlorinated Biphecyls (PCBs), polybrominated diphenyl ethers (PBDEs)	Longitudinal	Higher persistent organic pollutants (POP) exposure levels were associated with lower task-related functional brain activity in the inferior frontal gyrus and right anterior insula.
England-Mason et al. [203] Canada	76 mother-child pairs of Alberta Pregnancy Outcomes and Nutrition (APrON) study	4.4 (0.8) 51.3%	Phthalate	Longitudinal	Higher maternal prenatal phthalate concentration were associated with mean diffusivity in right inferior fronto-occipital fasciculus, right pyramidal fibres, bilateral uncinate fasciculus; and fractional anisotrophy in left inferior longitudinal fasciculus.

Exposure to toxins, whether environmental or chemical, can directly affect neural systems.

Higher arsenic exposure was associated with higher GMV in the IFG and lower GMV in the right inferior temporal cortex, right rostral ACC, and left insula [118]. Higher lead exposure in children was associated with smaller overall cortical volume and surface [119], particularly in the corpus callosum [120]. This is in line with studies on adults with high lead exposure in childhood, showing smaller cortical surface area and smaller hippocampal volume [121], and current exposure associated with smaller GMV in the cingulate gyri, insula, and corpus callosum [122].

Finally, alterations in functional activity and connectivity, including the insula, ACC, and hippocampus, could be observed for arsenic exposure [118]. For pesticides exposure lower FC in the left IFG and bilateral superior frontal gyrus was observed, during a behavioural inhibition task [123]. Aside the fact that more studies are needed, the existing ones provide similar evidence as also found for the effect of psychosocial adverse events, namely a complex interaction of exposure type, and age, on brain and behaviour.

## Perspective

**Fig. 1:**
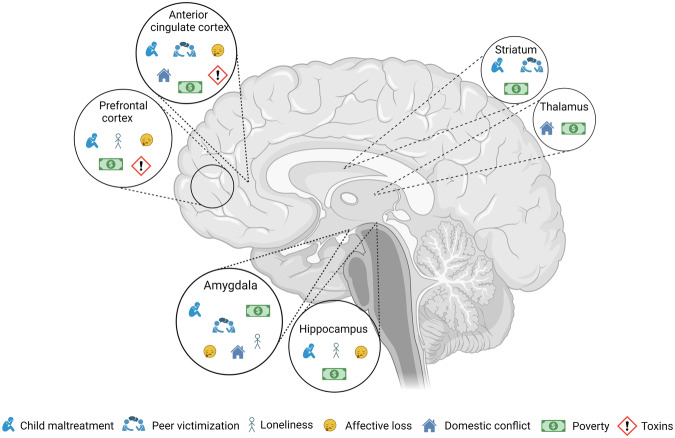
Allostatic load. This schematic illustration depicts the interconnectedness between the impact of various adversities on selected brain regions. Although each adversity may have distinct manifestations, they converge on common brain regions. Understanding the cumulative effects of these adversities on the brain can provide valuable insights into allostatic load.

Each form of adversity leaves its mark on the brain, affecting multiple regions. Across various forms of adversity, some common areas emerge (Fig. 1). This suggests that repeated and/or simultaneous occurrence of adverse life events may exacerbate allostasis, resulting in a cumulative impact on the neurobiology of specific brain regions. The exact nature of this cumulative impact however remains elusive.

The amygdala, hippocampus, prefrontal cortex and ACC are among the key regions that consistently show alterations in response to different adversities. These regions play crucial roles in emotional regulation, memory, and decision making, and their dysregulation has been linked to poor mental health outcomes [124, 125]. For example, the mechanisms of emotions, memory, and cognitive appraisal are interconnected in the amygdala and hippocampus, spanning from perception to reasoning. The amygdala-hippocampus is associated with two distinct memory systems, which interact with each other in emotional contexts. More specifically, the amygdala influences hippocampal-dependent memories, particularly episodic memory related to emotional stimuli. Conversely, the hippocampus can impact the response of the amygdala when encountering emotional stimuli. Thus, the amygdala and hippocampus modulate emotional memory processes, demonstrating their intertwined role in cognitive and emotional functioning [126]. Individuals experiencing alterations in these systems due to adversity may struggle with suppressing irrelevant aversive information [32, 65], impacting their emotional reactivity. Consequently, when faced with another adverse event, their response to the effects of such experiences may be heightened.

### Cumulative effects

The effects of various adversities are mostly studied in isolation, despite the interconnected nature of their impact. Investigations have examined either a single or limited number of exposures [127, 128], often using simple sum scores to access cumulative effects [129–131]. To unravel the biological underpinning of the combined effects of multiple adversities and understand if these effects are cumulative and/or synergistic, data-driven approaches can be explored. Recently, there have been some emerging but fragmented attempts of using data-driven approaches including machine learning [132], factor analysis [133] and clustering [131]. While on the one hand, these studies provide proof-of-concept for the benefits of employing a comprehensive and entirely data-driven approach to unravel the complex associations between diverse adversities and neurobiology. On the other hand, in 2 of these 3 studies, adversity sum score was taken.

Adversity itself exhibits considerable heterogeneity, further complicated by co-occurrence and chronicity. It is also important to acknowledge the heterogeneity underlying neuronal profiles, as different adversities will have common as well as varying effects on different individuals. Data-driven methodologies can aid in the development of conceptual models to achieve a comprehensive understanding of these intricacies. To achieve this, we can integrate profiles from multiple markers and across different modalities to characterise multivariate profiles of adversity. In practice, there may exist multiple partially overlapping risk profiles that operate differently in different individuals.

### Risk and resilience

Regions that are susceptible to the effects of adversity might experience exacerbated impairment with each hit, occurring simultaneously or across lifespan. This suggests a compounding effect, wherein the negative impact on neural structure and functioning is amplified with each subsequent exposure. For example, the presence of two or more early-life events worsened the age-related decline in hippocampus and amygdala volume [130]. In parallel, there is a growing body of literature on adaption-based approach to resilience (or hidden talents) that highlights the presence of intact or even enhanced social, cognitive, and affective skills among individuals who have experienced high levels of adversity [134]. These alternative perspectives emphasise the adaptive nature of certain phenotypes like attention, perception, learning, memory, and problem solving that emerge following adversity. They acknowledge that individuals who have experienced adversity may develop unique traits, skills, or coping mechanisms that can be beneficial in navigating challenging situations [135, 136]. For example, enhanced amygdala reactivity resulting from early adversity has been associated with improved goal-directed behaviour in situations where the goal aligns with threat-detection [137].

The impact of adversity can thus lead to distinct outcomes, with some individuals being categorised as at-risk and others as stress-adapted [136]. These observations underscore the heterogeneity in the impact of adversity, emphasising the need to shift from group-level inferences to individual-level predictions. By recognising the diverse and individualised responses to adversity, we can better understand the complex interplay between environmental exposures, neural mechanisms, and mental health outcomes.

### Psychopathology

Our review focused on investigating the effects of adversity on the brain, through an examination of population-based studies. Through this approach, we have summarised impairments in various brain regions and networks that were consistently associated with adversity. These neurobiological changes may have significant implications for psychiatry, potentially increasing an individual’s vulnerability to developing mental health disorders [138, 139]. For example: childhood adversity is associated with the onset of over 40% of childhood psychiatric disorders and more than 25% of adult psychiatric disorders [138]. Additionally, the observed variability and severity of symptoms or progression of the disease and comorbidity could potentially be attributed to brain changes from prior experiences of adversity. This has been reported for bipolar disorder [140], depression [141], conduct disorder [142], obsessive-compulsive disorder [143] and substance use [144]. Longitudinal studies also support these causal effects of adversity on psychopathology via brain changes. Blunted activation in the right amygdala associated with childhood adversity mediated its link with later externalising symptomatology [145]. In another study, changes in resting-state functional connectivity associated with childhood maltreatment [146] mediated the relationship with depression. And poverty was found to be associated with changes in hippocampal-amygdala connectivity, which also led to negative mood symptoms [147]. These findings highlight the implications of neurobiological changes resulting from adversity in psychopathology. By utilising multivariate predictive machine learning techniques, researchers can extend their investigations to make predictions regarding the initiation, progression, and outcomes of various illnesses. This approach holds promise for advancing precision medicine and offering valuable insights into tailored prevention and intervention approaches at the individual level.

## Future directions

Most studies we reviewed have principally identified group level associations with biological markers, generally in isolation from one another. While this provides an important first step in understanding the effects of adverse life events on the brain, we identify several key goals that we consider are necessary for the field to move towards a comprehensive understanding of real-life environmental impact, which can all leverage advances made possible by the increasing availability of big data cohorts.

### Unveiling multifaceted insights

To adequately capture and analyse higher-order interactions of highly collinear factors, it is essential to have large sample sizes to achieve sufficient statistical power [148, 149]. Large datasets enable the detection of subtle effects that may not reach statistical significance in smaller samples. Further, they ensure that the effects being investigated are robust and reproducible, allowing for more accurate and meaningful conclusions to be drawn from the data [148, 149]. We have specifically reviewed large samples, where possible, however most of these studies have used a traditional research approach, primarily providing descriptive findings. These studies have either examined associations between specific variables or have compared groups exposed to adversity with groups not exposed. While these approaches have provided foundational knowledge, there is a need to move beyond group-level effects towards understanding individual-level differences.

Machine learning methodologies offer a pathway for this transition from univariate associations to multivariate predictions. The utilisation of machine learning approaches to make predictions and separate groups is widespread in biological psychiatry [150] and such multivariate approaches also hold promise for integrating and understanding the cumulative effects of multiple adversities, more effectively than simple sum score methods. These methods are specifically designed to analyse multidimensional data, allowing researchers to uncover multivariate patterns that may not be readily apparent using traditional sum-score approaches. Data-driven approaches including supervised machine learning approaches [151, 152] and multivariate regression methods [153] such as partial least squares [118, 154] are emerging, and their potential is only beginning to be explored [155]. These techniques offer valuable tools for identifying latent variables within complex datasets, categorising individuals based on their unique profiles, integrating diverse sources of data, and finally facilitating prediction.

Another key goal for the field to move forward is developing optimal approaches for parsing heterogeneity, for which many approaches have been proposed in the field of psychiatry [156]. Parsing heterogeneity refers to the process of systematically analysing distinct sources of variability within a given dataset. By dissecting and categorising different sources of variability, researchers can gain a more intricate understanding of how various factors contribute to the overall outcomes observed. To do so, heterogeneity can be parsed at the variable level (i.e., to yield symptom groups or latent profiles) or at the individual level (to yield subtypes), which have been classically approached using variants of factor analysis [157] and clustering [158] respectively. To further parse heterogeneity approaches such as canonical correlation analysis [159], normative modelling [160] and anomaly detection methods [161, 162] can be explored. For example: In a recently published paper from our group, Holz et al. [163] employed a voxel-wise normative modelling approach to quantitatively assess heterogeneity in adversity effects. To estimate a pattern of regional deviations from typical brain structure for each participant, normative probability maps (NPM) were derived. Further, dice coefficients were calculated to assess the contribution of each adversity. This approach considered the correlated nature of adversities and helped explore both independent and combined long-term effects. Consequently, we found distinct neuroanatomical trajectories associated with specific adversities, indicating accelerated or delayed development in specific brain regions. Table 5 provides a brief overview of various computational approaches. For more details on statistical methodologies, we refer interested readers to Alpaydin [164] and Marquand et al. [154].

**Table 5:** Computational methods to parse heterogeneity.

Type	Description	Methods
Unsupervised learning approaches	Identify data components explaining maximal variance.	- Principal Component Analysis (PCA) - Independent Component Analysis (ICA) for neural data (specially fMRI).
Supervised learning approaches	Identifying factors that contribute to a particular outcome.	- Linear Discriminant Analysis (LDA) to discriminate between conditions. - Support vector machines (SVMs): • Linear SVM: when data are linearly separable. • Kernel SVM: handle non-linear mappings between features and outcomes. -Decision tree: A hierarchical model that makes decisions based on a series of conditions or features. -Random Forest: Merges multiple decision trees predictions to enhance accuracy and reduce overfitting. -The k-nearest neighbors (kNN), a nonlinear classifier. -Neural networks: • Convolutional neural networks (CNN); useful for data that contain spatial structure (e.g.: images) • Recurrent Neural Networks (RNNs), including Long Short-Term Memory (LSTM) networks, are ideally suited for handling longitudinal data. • Graph Neural Networks (GNNs) are specialised for analysing graph-structured data.
Doubly multivariate regression	Extract a series of components from multiple sets or views of data that maximally co-vary with one another.	- Canonical Correlation Analysis (CCA) to maximise the correlation among views of the data. - Partial Least Squares (PLS) to identify components with maximum covariance.
Bayesian networks	Probabilistic graphical models build on cause-and-effect relationships.	- Naïve Bayes: A probabilistic classifier assuming strong independence between features. - Dynamic Bayesian network is capable of modelling relationships in time-series data.
Normative Modelling	Identification of deviations from typical patterns in data.	All normative modelling methods aim to model centiles of variation in large cohort data, aiming to use these to make individualised predictions. - Hierarchical Bayesian regression (HBR) - Bayesian Linear regression (BLR) - Generalised additive models for location scale and shape (GAMLSS) - Gaussian process regression (GPR)

### Cohort synergy

To study individual level inferences, longitudinal designs that enable the investigation of within-individual change over time, are considered gold standard [165, 166]. The utilisation of longitudinal models to delineate trajectories would help elucidate the specific nature of deviations caused by adversity, including potential delays or accelerations in development. Further, adversity’s effects may differ depending on the timing of exposure. Despite evidence for the same [27, 28, 111], few studies have examined sensitive periods. Understanding the underlying mechanisms and what is biologically embedded during sensitive periods is crucial for comprehending how experiences shape neurobehavioral outcomes. Incorporating insights from formal modelling can help bridge these gaps [167]. Finally, questionnaires could be modified to capture not only the timings of adversity but also their duration, for a more intricate understanding of exposure. This information could serve as the foundation for conducting comparative analyses across different age groups, enabling the discernment of age-, duration- related variations in the impact of adverse experiences on brain.

While longitudinal tracking is a viable alternative, it is necessary to have a sizable group of children across a wide age range who have been exposed to adversity, as well as those who have not. To recruit and track a large sample is a challenge and consequently, the next crucial step is to develop methods for data pooling across different labs. By pooling cohorts and data, the size of datasets can be significantly increased. Machine learning approaches can then be applied to understand within population differences (e.g.: SES may vary across cohorts) and between population similarities (e.g.: loneliness). Further, data can be pooled across cohorts from different timepoints in an accelerated longitudinal design [168] with structural equation models estimate to converge multiple pieces into a single latent growth or specific latent classes by age [169]. Additionally, adopting age as a proxy for time, as in pseudo-longitudinal designs [170], offers a valuable strategy. Pseudo-longitudinal designs enable the exploration of temporal trends without the extended time commitment of traditional longitudinal research.

To address the challenge of binding data from different cohorts, neuroimaging studies can utilise statistical harmonisation techniques such as ComBat [171]. ComBat is a widely used method in neuroimaging research that aims to reduce batch (site/cohort) effects across different datasets but can also introduce bias [172]. Alternatively normative modelling can be used to accommodate site effects by providing a common reference to bind different samples together, thereby facilitating pooling of data for analysis [173]. Harmonisation of other kinds of data bring additional challenges such as aligning different measurements of the same construct. However, efforts have been initiated in these directions [174] and will be a major focus within the environMENTAL consortium (Schumann et al. [175]). This project aims to leverage existing cohorts from the past two decades, to enhance the efficiency of population neuroscience research, by harmonising the data that has already been collected. Also, cohorts (where participants can be re-contacted) will be enriched to address measurement issues and achieve the necessary depth of phenotyping for data-driven models.

### Unravelling specific mechanisms

Big data studies have the advantage of large sample sizes, however, they may not capture the prevalence of some adversities (e.g.: institutional rearing). Thus, meta-analysis or multi-site collaborative studies are also important. The integration of information across centres and modalities might contribute to systematic mapping of neuronal plasticity. Further, certain adversities may exhibit small effect sizes individually but converge on similar outcomes (e.g.: toxins). Although these effects may be small, it is crucial to consider them for a nuanced understanding of the overall cumulative impact. A useful analogy can be drawn from genetics, where the aggregation of small genetic polymorphisms is compared to a watershed phenomenon [176]. As these genetic variations accumulate downstream, they eventually manifest in the syndromic expression of a disorder. Similarly, studying the effects of multiple diverse adversities can provide a comprehensive understanding of their combined influence.

Lastly, while large-scale studies provide generalisability, they may lack the detailed investigation of specific mechanisms. On the other hand, smaller experimental studies have the advantage of greater control, to delve into intricate processes. Thus, a combination of both large-scale and smaller experimental studies can enrich our understanding, with the former providing broader insights and the latter being more focussed and detailed.

## Conclusion

The complexities of cumulative adversity and its effects on the brain pose significant challenges in terms of unravelling the underlying mechanisms and establishing comprehensive models. The heterogeneity in individuals’ experiences of adversity, the timing and duration of exposures, and the potential moderating factors all contribute to the complexity of the phenomenon. An examination of the current state of the field underscores the significance of conducting biological investigations on large-scale samples. Linking adversity to underlying biological mechanisms can help gain insights into the unique profiles and needs of individuals. Moreover, the utilisation of advanced computational approaches to disentangle heterogeneity and the combined effects of diverse adversities hold promise in this area. Continuous improvement of conceptual models, integrating insights gained from such research endeavours, will be critical for advancing knowledge and facilitating the translation to understanding data-driven sources of individual variance.
